# Improving the accessibility to public schools in urban areas of developing countries through a location model and an analytical framework

**DOI:** 10.1371/journal.pone.0262520

**Published:** 2022-01-12

**Authors:** Jesica de Armas, Helena Ramalhinho, Marta Reynal-Querol

**Affiliations:** 1 Department of Economics and Business, Universitat Pompeu Fabra, Barcelona, Spain; 2 UPF Barcelona School of Management, Barcelona, Spain; 3 Barcelona Graduate School of Economics, Barcelona, Spain; 4 Institución Catalana de Investigación y Estudios Avanzados, Barcelona, Spain; 5 Institute of Political Economy and Governance, Barcelona, Spain; University of Zilina, SLOVAKIA

## Abstract

The location of primary public schools in urban areas of developing countries is the focus of this study. In such areas, new schools and modification of the current schools are required, and this process should be developed using rational and broad supporting tools for decision makers, such as optimization models. We propose a realistic coverage location model and a framework to analyze the location of schools. Our approach considers the existing schools and their resizing, the best locations of the new schools that may have different capacities, population coverage, walking distances and budget provisions for building and updating schools. As a case study, we assess the current primary school network in Ciudad Benito Juarez to provide managerial insights. Through the proposed framework, we analyze the current locations of schools and decisions to be made by considering future scenarios in different time periods. The proposed model is quite flexible and easy to adapt to new considerations, allowing it to be applied to regions in developing countries under similar conditions.

## Introduction

The impact of the educational system on the growth and development of a country is well known. Access to education, particularly at initial stages of education, leads to added value and employment growth, an increase in the GDP, and reduction of social and economic inequalities [1–4]. Additionally, access to education is one of the United Nations Millennium Development Goals [5]. Thus, the education public services provided by any government are surely one of the most important public services, especially considering the great need for investment and the impacts on society, economic growth, inequality reduction, and development of the country. In the development economics literature, [6] mentions that “Nations cannot be developed without investing in education”. [7] states that “education is a key factor in countries’ development as it is proven to be effective in fighting poverty, creating more opportunities for labour market participation, increasing economic growth rate, allocating resources within the common environmental contests and ultimately, achieving sustainable socio-economic development”. [8] argues that “education in every sense is one of the fundamental factors of development”.

Since the accessibility to schools improves the level of education of a population, it impacts on the development of a region. Understandably, the education system needs physical infrastructure, i.e., schools, to provide the educational services. The location of these schools affects access to education, particularly in developing countries where private and public transportation options are limited or not easily accessible by the whole population [9]. Some studies support the impact of the location of schools on attendance. [10] assesses the relevance of supply and demand side factors in determining rural primary school enrollment in Mozambique. This author finds that school availability significantly impacts enrollment rates. Estimates suggest that reducing the travel time to the nearest school leads to an increase of 17–20% in enrollment rates. Policy simulations show that building more schools or increasing adult literacy will exert a larger impact on enrollment rates than interventions related to raise household income. Later, [11] documents the average travel distance to school for Nigerian students in primary and secondary schools, as well as its impact on attendance. The author gathers data through a questionnaire administered to 166 schools. The study reveals that the majority of the students have to travel between 1 and 5 kilometers to school. Moreover, more than 85% of all schools interviewed reported that the distance traveled to school every day by both primary and secondary school students exerts a substantial adverse effect on their attendance. Academic performance is also susceptible to improvement through the optimized location of schools. [12] evaluate a village-based randomized school program in Afghanistan to identify the effect of placing a school within a village on children’s academic performance. The authors find a positive impact on academic participation and performance among all children, particularly for girls.

To plan the location of schools properly, there are several aspects to take into account, for example, population growth and public investment level, or even community-based issues, such as local impact, diverse populations or spatial concerns [13]. Since education is a public service, these decisions should be transparent and rational. In this work, we apply operations research (OR) location models and algorithms to find the best locations of schools, taking into account the spatial distribution of students, the location of existing schools, the maximum walking distance between the students’ neighborhoods and the schools, investment levels, etc. Since the number of possible planning solutions for this location problem is exponential, optimization models are indispensable decision-making tools to enable evidence- and data-based decisions to be made. OR has contributed widely in fields such as developing countries [14] or community-based organizations [13, 15]. This discipline gives support to making decisions about education, public safety, human and social services, community and economic development, and environmental preservation.

Many optimization models can be applied within public facility planning processes. Certainly, OR location models are one of the most used [16]. The aim of these models is basically to determine the most efficient locations for all kinds of facilities according to some objective or objectives, such as minimizing the cost reduction or maximizing accessibility by the population.

The focus of the present work is on the location of public schools in relatively poor areas that are likely to see substantial population increases in coming years. We consider that the public schools are equally attractive and have the same quality, similar teaching, and comparable recreation spaces, tradition, and pupils from the same social stratum, which is the premise of a public education system. In these regions, private schools and private or public transportation are not commonly available for the majority of the population. Thus, the school choice or the quality of public transport [17–19] fall outside the scope of this work. In this reality, walking from home to school is the most common method of transportation and, therefore, the location of the school makes a greater impact on the accessibility by the population than in other regions with private school choice and other transportation means.

An example of this kind of area is Ciudad Benito Juárez, a city located in the eastern part of the Monterrey metropolitan area in the state of Nuevo León, Mexico. This city had a 2010 census population of 150,000 inhabitants and is the seventh largest city in Nuevo León. The census in 2000 reported 13,230 inhabitants, and in 1990, there were 9,151 inhabitants. This means that in ten years (2000-2010), there was a population increase of more than 1000%. Thus, this city has been practically built since 2000; i.e., all of the schools and public services have been incorporated from that date onward. Additionally, the population growth forecasts estimate that the population will reach 270,000 inhabitants by 2030 [20].

This work assesses the current primary school network in Ciudad Benito Juarez through a realistic and novel coverage location model and a framework of analysis. In the first step, the current scenario is analyzed to obtain the current coverage values at different walking distances. In the second step, decisions about expanding the current schools to improve the coverage are analyzed while considering different budgets and walking distances. In the third step, the possibility of building new schools is analyzed, in addition to expanding the current schools, while considering different budgets, walking distances and numbers of new schools. The fourth step consists of comparing and analyzing the differences between the current locations of schools and the optimal locations if we start from an empty map and build the same number of schools with the same budget. This step can provide food for thought and valuable insight about how far the current location is from the optimal location, why some bad decisions were made in the past, how it would have been avoided, and the usefulness of our approach for the future. In the fifth step, population projections for the ensuing years are considered to analyze future scenarios. Decisions about the optimal location of new schools and resizing of the current schools to reach a suitable coverage are analyzed for different time periods. This last step allows evaluation of decisions about the future locations of schools.

The proposed framework can be applied to any region subject to an intense population growth process with a great need of proactive measures to avoid an unsatisfactory public education system. The mathematical model will provide solutions for the location of new schools, the resizing of the current schools, and the assignment of the neighborhoods to the schools to maximize the accessibility of the student population. This approach is quite flexible and easy to adapt to new considerations, allowing its application to regions in developing countries under similar conditions or to regions after suffering a natural disaster and with the pressing need to rebuild their school systems. The overall methodological approach proposed in this work can become a general decision-making tool for decision makers, usually politicians, to make rational, transparent and fair decisions. In fact, a dialogue between the political establishment and technical evaluations, and even community collaboration and engagement [13] should be considered when making decision that affect the whole society. In this way, politicians, OR specialists and communities may converge to make better decisions that can make a great impact on society. The documentary ‘On the way to school’ [21] illustrates the sorts of problems that this study addresses.

Therefore, the main contributions of this research can be summarized as follows. First, we propose an optimization model that includes many important features described in previous works on school location at the same time. We present a general framework that can be used to develop an analysis of the school location in any region under intense population growth. We then present an extensive analysis on Ciudad Benito Juárez to exemplify this methodology. Finally, the proposed methodology can help to improve access to education in developing regions.

## Related works

In this paper, we focus on the location of public primary schools in relatively poor areas with a sharp population increase tendency (population growth rate higher than 2%). The walking autonomy a young student has in these areas needs to be considered when dealing with this kind of decisions. This is the case of Ciudad Benito Juarez. Therefore, we establish maximum distances and focus on the fact that the number of students under this assumption has to be maximized. Regardless, every student should attend one school, and therefore, the capacity of schools must be sufficient to satisfy the demand. We take into account the existing schools, along with their capacities, since it would be unrealistic to ignore their existence. Nevertheless, we consider the possibility of expanding them and building new schools based on the sharp population increase tendency in these areas. The new schools could have different capacities, which represents an additional decision to make. Finally, the budget involved in these expansions and new construction is quite relevant especially in the context of developing countries, where the budget is low (very restrictive) and small deviations can make a difference for many people. Therefore, it has been considered in our approach.

Several reviews covering location problems have been developed in the literature. [22] provides a comprehensive review of models, solutions, and applications related to the coverage problem in facility location after [23]. Later, [24] presents a more recent overview of the maximal covering location problem, emphasizing the application, solution, evolution, and extension of it. It is worth noting that the areas of application of location models are very extensive (telecommunications, healthcare, geographic information systems, rapid transit networks, etc.) and it is out of the scope of this work to analyse them. This is the reason why we focus on school location in the following.

Most of the state-of-the-art approaches about school location use maximum covering and the p-median models. Notice that the first approach consists of deciding the number and the locations of the facilities, maximizing the total demand coverage within a maximal service distance or time from a facility, while the p-median problem consists of deciding the locations of p facilities to minimize the weighted average distance (or cost) between demand nodes and the nearest of the selected facilities. As stated in the introduction, one important factor in improving the access education and the enrolment and attendance to school, in particular in developing countries, is distance to school since in many of these countries the students access the school by walking. Therefore, we believe that the maximum covering problem represents in a more adequate way the objective of improving the accessibility to public schools in developing countries, than the weighted average distance alone.

One of the earliest works focusing on school location was published by [25]. The authors propose a maximum coverage model to locate a fixed number of schools and maximize the population covered within a maximum travel distance. School capacity is not explicitly considered and only one school size is assumed. In a more general context, [26] deals with the capacitated version of the maximum coverage problem and proposes different models. These models do not consider existing facilities, the possibility of expanding them, different capacities or budgets.

Later relevant works in school location tend to use models based on the p-median. [27] applies it to determine the optimal location of schools in two counties of Rio de Janeiro. Again, the authors do not consider the capacity explicitly in the model, which is also performed by [28] in the area of Dakar, Senegal. To address this issue, [29] inserts different capacities and solve the problem for Vitoria, a state capital located in the southeastern region of Brazil. [30, 31] also consider different school capacities in regions of Portugal and Malaysia, respectively, and extend the model to include different levels of education (primary schools, secondary schools, high schools, etc.), solving the hierarchical p-median problem. None of these works account for the existing locations of the actual schools, the resizing of the existing schools or the different capacities of schools. They solve the problem by assuming that the area has no schools and then compare the solution with the current scenario to indicate whether some schools need to be closed or opened.

In this sense, our work is more realistic since the current schools are taken into account in the model. Thus, it is possible to add new schools and modify the capacity of the existing schools. [32] addresses a similar problem using two different models: the capacitated p-median model and the maximum covering location model. Solutions obtained by the two models for Guaratiba, Rio de Janeiro, need to be compared in order to find a common solution since each model by itself does not consider the whole set of features and constraints of the problem that need to be solved. Additionally, although results show the current schools, the actual schools are not explicitly considered in the models or the solutions obtained. [33] deals with the problem where current schools are taken into account: these schools can be closed or resized, and new schools can be opened. In this regard, this work is closer to our proposal. Nonetheless, the demand is assumed to be concentrated in a given number of ‘centers’, municipalities, towns of a region or neighbourhoods, which corresponds to a high level of aggregation. In this regard, we consider census blocks, i.e., the minimum level of aggregation that data allows, which usually results in better estimations. Additionally, in that work distances are not considered since the objective is to minimize the cost. Therefore, this is not a coverage problem like the one that we address, where the distance plays an important role. The work focuses on general countries and does not take into account the characteristics of developing nations.

Similarly, [34] considers the current schools and the possibility of opening, closing, or resizing them. The focus is on a rural case that includes the peculiarity of scarce demand in some places, multigrade schools, and schools with only some grades. The authors reduce the potential locations to a set of candidates, and it is possible to exceed the capacity of schools. Although this model includes many of the attributes that we consider in our problem, it presents some limitations for our application. We consider walking distances and the substantial adverse effect of large distances [32, 35] on enrollment, attendance and academic performance in the context of developing countries [10–12]. Therefore, maximizing the coverage at a maximum distance is the focus of our proposal. However, this model focuses on minimizing costs, and coverage is not relevant. The purpose is different due to the scope of application.

There are some other relevant proposals in the literature about school location, although the purposes and assumptions are different from our proposal. Just to mention some of these approaches, [36, 37] focus on the metropolitan environment and take into account bus transportation, socioeconomic status, and surplus real property management; in this regard, [38] proposes an approach that deals with the school location problem as well as the school bus routing and shift programming problem. [39–43] address location problems while considering that students can choose the school that they wish to attend. This is common when private schools are available and is not typical in developing countries, where the allocation of students to schools is mandatory and usually only to the closest school. [9] addresses the use of public transportation and assess the school location impact on the educational inequalities. Additionally, [44, 45] consider the appropriate racial balance levels at each school.

Particularly with regard to the differences between rural and urban location of schools, several studies indicate that there exist differences related to school infrastructure, geographic distance, type of population, teacher quality, learning outcomes and education expectation, access/transportation to school and socio-economic factors [46–49]. The proposed model and framework can be applied in both cases, with an adjustment of the parameters or data of the model depending on the type of area to be planned. Whenever the decision maker uses the framework proposed to locate schools, the following parameters can be different depending on whether the planned area is rural or urban: the total budget available, the geographical distance that impacts the maximum walking distance, number of new schools to be opened, and the different capacities. For example, the maximum walking distance and the capacities in rural areas can be different from the urban areas. Therefore, it is relevant that the decision maker has a good knowledge of the planning area before applying the framework. However, a strong aspect of the proposal in this work is that the model and framework can be applied to different areas and scenarios.

Table 1 allows to conclude this review with a useful overview of the differences between our proposal and the state-of-the-art approaches closest to it. The first three columns contain the reference to the particular work, the name of the location problem addressed and the objective function considered. The next columns indicate whether or not the mathematical model in the proposal considers the following features: capacity of schools, current existing schools, resizing of schools, schools with different capacities, population coverage, distances between students and schools, and budget to build/resize.

**Table 1:** Comparison of approaches.

Paper	Location Problem	Objective	f1	f2	f3	f4	f5	f6	f7
[25]	Maximal coverage	Coverage maximization	✘	✘	✘	✘	✔	✔	✘
[26]	Capacitated maximal coverage	QoS maximization	✔	✘	✘	✘	✔	✔	✘
[27]	P-median	Distance minimization	✘	✘	✘	✘	✘	✔	✘
[33]	Dynamic Modular Capacitated Facility Location	Total discounted (socioeconomic) cost minimization	✔	✔	✔	✔	✘	✘	✔
[29]	P-median	Distance minimization	✔	✘	✘	✘	✘	✘	✘
[30]	Hierarchical p-median	Travel cost minimization	✔	✘	✘	✔	✘	✔	✘
[28]	P-median	Distance minimization	✘	✘	✘	✘	✘	✔	✘
[34]	Capacitated Facility Location	Total annual cost minimization	✔	✔	✔	✔	✘	✔	✔
[31]	Hierarchical p-median	Distance minimization	✔	✘	✘	✘	✘	✔	✘
[32]	Capacitated p-median and maximal coverage	Distance minimization/Coverage maximization	✔	✘	✘	✘	✔	✔	✘
This approach	Capacitated maximal coverage	Coverage maximization	✔	✔	✔	✔	✔	✔	✔

f1: capacity of schools, f2: current existing schools, f3: resizing of schools, f4: schools with different capacities, f5: population coverage, f6: distances between students and schools, f7: budget to build/resize

Therefore, this work contributes to the school location modeling in developing countries as follows: First, a school location model close to reality is proposed while considering the existing schools and their resizing, the best locations of the new schools, which may have different capacities or population coverage, relevant walking distances in developing countries, and budget provisions for building and updating schools. These assumptions have not been simultaneously considered in any previous school location model, to the best of our knowledge. We have not even found a location model in other field of application (using the compilation of works included in the surveys [22–24, 50], and relevant works not included in these sources) that takes these characteristics into account. Second, we demonstrate the application to a real case study with real data. This approach can be extended to other similar areas in the world. Third, an analytical framework to study the locations of schools in developing areas is presented to help politicians or public service managers to make data-based decisions that are more transparent and efficient, and consequently to positively impact society. Finally, the ease and flexibility of the model permit it to be adapted to solve different scenarios and to derive managerial insights in different situations, such as with respect to growing population, or even in the case of a natural disaster situation after which a completely new set of schools must be opened. Eventually, the model can be adapted to other public services such as healthcare, day care centers, or nursery schools, for example.

## Problem description, mathematical model and analytical framework

In this section, we describe the problem of locating public schools in a region facing significant population growth, which poses a serious challenge to providing education in proper conditions. As mentioned, the problem is motivated by a real case in Ciudad Benito Juárez. Since the population has grown by 1000% in this city between 2000 and 2010, and is expected to keep growing, it is very important to properly determine the locations of future public primary schools to be able to provide effective education services. One relevant aspect in order to determine their locations is the distance from the students’ homes to their school, since long distances are not desirable. Obviously, the current primary school locations need to be considered since they are built and operational, although we consider the possibility of resizing them.

Thus, the idea behind our proposal is obtaining the locations of new schools, their capacities, and the resizing of the current schools (if needed) so that the number of students who attend a school within a maximum walking distance is maximized. If this number is depicted as a percentage with respect to the total number of students, then this is known as the coverage of the plan of school locations. This coverage depends on the given distance, the budget available to build new schools and expand the current schools, and the number of new schools considered.

We propose an integer linear programming (ILP) model for the public school location problem. We use the following notation for the sets, parameters, and variables:

Sets:

-
N={1,…,N} : Set of blocks
-
L={1,…,L} : Set of blocks without schools where schools can be built
-
M={1,…,M} : Set of blocks with schools (where schools can be resized)
-
T=L∪M : Set of blocks where schools can be located
-
K={1,…,K} : Set of indexes of school capacities, which are sorted in an increasing manner

Parameters:

- *B*: Total budget
- *D*_*max*_: Maximum walking distance
- *P*: Number of new schools to be opened
- *a*_*j*_: Index of the current capacity of a school in block j∈M
- *c*_*k*_: Capacity k∈K
- *d*_*ij*_: Distance between the centroid in block i∈N and centroid in block j∈T
- *e*_*jk*_: Expenses of building a school in block j∈L with capacity *c*_*k*_
- *u*_*jk*_: Expenses of updating a school in block j∈M to capacity *c*_*k*_
- *r*_*ij*_: 1 if *d*_*ij*_ ≤ *D*_*max*_; 0 otherwise
- *w*_*i*_: Weight at block i∈N (population)

Variables:

- *x*_*ij*_ = 1 if block i∈N is assigned to a school in block j∈T; 0 otherwise
- *y*_*jk*_ = 1 if a school in block j∈T has capacity *c*_*k*_; 0 otherwise

The main objective of the model is to maximize the number of students who are assigned to a school that is within the maximum desired distance subject to the following criteria: i) students assigned to schools do not cause the capacity to be exceeded, ii) the budget constraint is verified, and iii) current schools are already built, so we only considered that their capacity can be maintained or increased. Notice that it is assumed that only one school can exist per block. Thus, the following ILP model formulates the public school location problem:

$$\begin{array}{cc} & \text{Maximize}\sum_{i\in N}\sum_{j\in T}r_{ij}w_{i}x_{ij} \end{array}$$ (1)

$$\begin{array}{cccccc} & \text{subject}\;\text{to}\\ & \sum_{j\in T}x_{ij}=1 & & & & \forall i\in N \end{array}$$ (2)

$$\begin{array}{cccccc} & \sum_{i\in N}r_{ij}w_{i}x_{ij}\leq \sum_{k\in K}c_{k}y_{jk} & & & & \forall j\in T \end{array}$$ (3)

$$\begin{array}{cc} & \sum_{j\in L}\sum_{k\in K}y_{jk}=P \end{array}$$ (4)

$$\begin{array}{cccccc} & \sum_{k\in K}y_{jk}\leq 1 & & & & \forall j\in L \end{array}$$ (5)

$$\begin{array}{cccccc} & \sum_{k=1}^{a_{j}-1}y_{jk}=0 & & & & \forall j\in M \end{array}$$ (6)

$$\begin{array}{cccccc} & \sum_{k=a_{j}}^{K}y_{jk}=1 & & & & \forall j\in M \end{array}$$ (7)

$$\begin{array}{cc} & \sum_{j\in L}\sum_{k\in K}e_{jk}y_{jk}+\sum_{j\in M}\sum_{k\in K}u_{jk}y_{jk}\leq B \end{array}$$ (8)

$$\begin{array}{cccccc} & x_{ij},y_{jk}=\left\{0,1\right\} & & & & \forall i\in N,j\in T,k\in K \end{array}$$ (9)

The objective function (1) consists of maximizing the total student population covered by *D*_*max*_ distance, using *P* new schools and the current schools. Constraint (2) ensures that every block is assigned to a block with a school. Constraint (3) indicates that if a school is located in block *j*, the student population assigned to this block must be less than or equal to its capacity. Constraint (4) limits the number of new schools over all school blocks and capacities to *P*. Constraint (5) limits to one the number of new schools per block. Constraints (6)–(7) ensure that the current schools are considered with their actual capacities or higher capacities. Constraint (8) takes into account that the total expenses needed to build the new *P* schools and update the current schools cannot be higher than the available budget. Finally, constraint (9) indicates that decision variables are binary.

Notice that if the expansion of current schools is not considered, constraint (7) is replaced by the following:

$$\begin{array}{cccccc} & y_{ja_{j}}=1 & & & & \forall j\in M \end{array}$$ (10)

$$\begin{array}{cccccc} & \sum_{k=a_{j}+1}^{K}y_{jk}=0 & & & & \forall j\in M \end{array}$$ (11)

It is worth mentioning that to the best of our knowledge, this is a new formulation of a coverage location problem that includes many real details and constraints that were not previously simultaneously addressed in the related literature. As previously shown in Table 1, there is not previous work in the literature that simultaneously considers the capacity of schools, the current existing schools, the possibility of resizing them, different school capacities, population coverage, distances between students and schools, and budget provisions to build/resize.

Once the problem has been mathematically modeled, we propose a framework to develop the analysis of the location of schools in any region in the context of an intense population growth process and with a great need for proactive measures to avoid the predicted collapse of the public education system. This framework is composed of the next steps:

1. **Analysis of the current state of school locations**.
  - Goal: Analyze the current scenario to obtain the current coverage at different walking distances.
  - Insight: This allows determination of the initial state and possible needs to improve. Although it may seem trivial, the current scenario greatly influences the following steps.
  - Procedure: The ILP model is run with *P* = 0, *B* = 0 and different maximum distances *D*_*max*_ to obtain the current coverage.
2. **Analysis of the expansions of current schools**.
  - Goal: Analyze the possibilities that the current school expansion may offer.
  - Insight: This allows for analysis of decisions about expanding the current schools to improve the coverage. This is a conservative option and has limitations. Nevertheless, it is important to understand the opportunities that the current locations of schools may offer.
  - Procedure: The ILP model is run with *P* = 0, different budgets *B* and maximum distances *D*_*max*_.
3. **Analysis of the locations of new schools**.
  - Goal: Analyze the possibility of building new schools in addition to expanding the current schools. Different budgets, walking distances and numbers of new schools are considered.
  - Insight: This allows decision makers to choose the option that best fits the needs and resources.
  - Procedure: The ILP model is run with different numbers of new schools *P*, budgets *B* and maximum distances *D*_*max*_.
4. **Comparative ab initio analysis of the scenarios**.
  - Goal: Analyze the differences between the current locations of schools and the optimal locations if we start from an empty map.
  - Insight: This step is key to understanding what would have been the best locations for the current schools and therefore, in the best of cases, what coverage would have been available.
  - Procedure: The ILP model is run with the same number of schools in the current scenario and the same budget invested in order to conduct a fair comparison. The input data must indicate that there are no initial schools located.
5. **Projections and location analysis for the future.**
  - Goal: Analyze future scenarios considering population projections during the ensuing years. Decisions about the optimal locations of new schools and resizing of the current schools to reach a suitable coverage are analyzed for different time periods.
  - Insight: This step allows evaluation of decisions about the locations of schools in the future.
  - Procedure: The ILP model is run with the initial scenario, different numbers of new schools *P*, different budgets *B* and the population projections for the next period. Definition of the target coverage at a maximum walking distance is important in order to make comparisons at this level. The output obtained is used as input for the next period with the next population projection.

### Data and modeling assumptions

**Fig 1:**
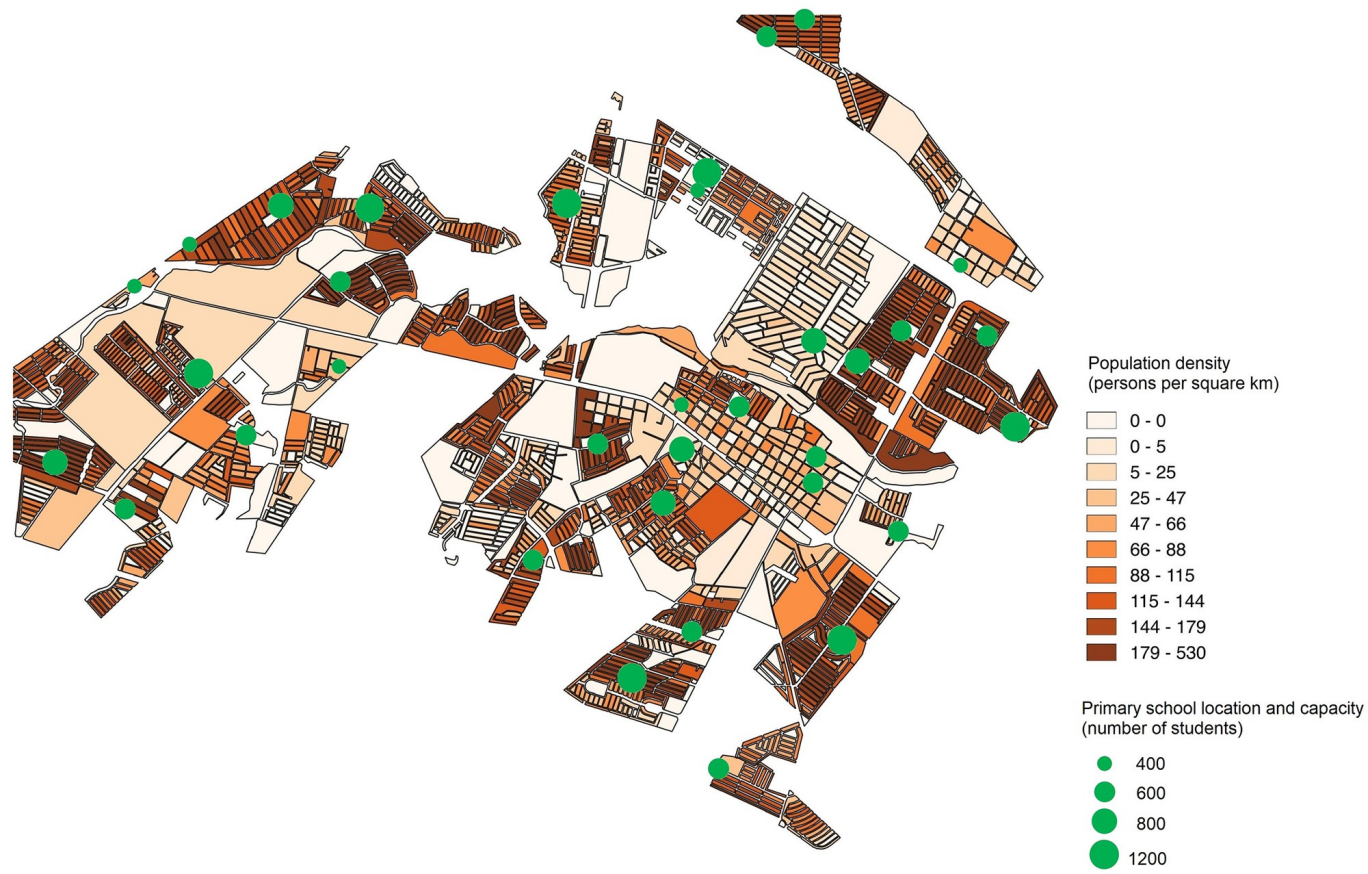
Population density and location of the 34 primary schools in Ciudad Benito Juárez in 2010.

As stated before, the aim of this work is to assess and improve the location of public primary schools in urban areas of developing countries through the use of the proposed ILP model. Ciudad Benito Juárez is used as a case study to demonstrate its usefulness as a decision-making tool. Fig 1 shows an overview of the city, indicating the locations of the 34 primary schools and the population density in 2010. In this city, there are 2041 census blocks. It is worth mentioning that we are assuming that a school can be built in any of the 2041 blocks. A detailed analysis of the area at a geographical, social and cultural levels would be needed, which is outside the scope of this work. However, if this is not possible in some blocks or it is not appropriate due to, for example, community restrictions [13], then it is acceptable to specify it in the input data since the model is quite flexible in addressing these issues. Here, the idea is to propose and evaluate a decision-making tool that can be applied in developing countries and to derive managerial insights in different situations.

Population data at block level have been obtained from the National Institute of Statistics and Geography in Mexico, INEGI. The latest available data are from 2010, which is the reason why we focus our study and the projections based on it on this year. Notice that we only consider primary-school-aged population to develop our study. Moreover, the number and capacities of schools in 2010 have been obtained. The regular capacities in Ciudad Benito Juarez are 400, 600, 800, and 1200 students. For 11 of the 34 schools, data about the capacity are not available, so the average capacity of the remaining schools has been calculated and fixed. This average is 750, but this is not a regular capacity in this area. As the regular capacities close to this value are 600 and 800, we set the capacity of half of these schools to 600 and the rest to 800.

It is worth-noting that depending on the country, or even the area, there are some education rules and regulations that determine the maximum capacity of a classroom in primary school (in number of students), and also some rules about the dimension (in number of classrooms) when an architectural plan for schools is considered. Therefore, school capacities in most of the regions cannot take any value, but a few discrete values. For example, [33] address a school location problem in Portugal where capacity modules of 150 students (25 students per classroom) are used, and therefore school capacities must be multiple of 150, moving from 450 to 1200. [42] deal with a case study in Germany and use low capacity levels, from 3 to 5, with 25 students per classroom. [29] solve the problem in Brazil and mention that the standard capacity of schools to be adopted there is 700 students. Thus, the possible school capacities depend on the particular place, but usually correspond to a discrete set of values.

Another important data consideration that is necessary to analyze the location of schools is the distance that each individual student must traverse from home to school and vice versa. However, this requires his/her precise residential address, and this information is not available due to safeguarding privacy. To overcome this impossibility, distances from each block centroid to its closest school block centroid have been determined. These distances along the road network have been calculated through the QGIS tool. Every student residing in a particular census block is supposed to live precisely on its centroid. This is a usual and reasonable approximation in urban studies.

Furthermore, with the aim of applying the proposed approach in this analysis, some budgets for building and expanding schools need to be fixed. On the one hand, no evidence has been found about different prices depending on the block inside the city. On the other hand, new construction and expansions do differ, with expansions being less expensive. Currently, three schools are being expanded, from 600 to 900 and 1200 students, and with budgets from 1,000,000 to 1,700,000 MXN$ (approximately 40,000-70,000€) [51], while new construction could double these figures.

Once all of this information about 2010 has been gathered, a study of the locations of public primary schools, their coverage values at different distances, their capacities, and the construction of new schools to improve the picture can be developed using the proposed approach. Additionally, based on these figures from 2010, projections of population growth have been calculated in order to assess the scenario in future five-year periods. According to projections [20], the expected growth from 2010 to 2030 is 80%, and we have deemed it homogeneous in terms of age in the absence of more relevant data. We divide this percentage homogeneously during the time horizon, so we consider 20% growth for each period of five years. Then, a comparative study has been carried out for 2015, 2020, 2025 and 2030 using these data.

Notice that in very recent updated data about population in 2020 [52], these 80% have been exceeded. This further strengthens the needs of the optimization framework that we propose in this kind of areas.

## Results and discussion

In this section, a set of experiments is presented with the aim of analyzing the location of public primary schools in Ciudad Benito Juarez in 2010 and future periods. The main objective of these experiments is to exemplify the application of the proposed framework to help decision makers to locate public schools in the best possible way in intensively growing population areas. We use the last available data (from 2010) and projections for each five-year period until 2030 to develop the following experiments according to each step in the proposed framework.

These experiments were possible owing to the implementation of the ILP model using Java SE 8 and the CPLEX optimizer 12.6.3 over the Netbeans IDE. The model was run in a cluster with CentOS 7.x x86_64, 27 computing nodes, 720 cores, and 7.4 TB of RAM. Despite the substantial capacity of this cluster, just one node with 128 GB of RAM was available for use. Maps have been generated using QGIS 2.18.14. For the initial experiments, the computing time was fixed as one hour, and results were obtained with a relative gap (i.e., the difference between the upper bound and the best solution obtained divided by the best solution obtained) below 1%. It is worth noting that this is a strategic problem, and therefore, it is not necessary to solve it in seconds or minutes. Additionally, we solve the problem for a city. If a larger area needs to be planned (e.g. a country), the most common way to deal with it is dividing it in smaller geographic areas [34].

### Analysis of the primary school locations in 2010

**Fig 2:**
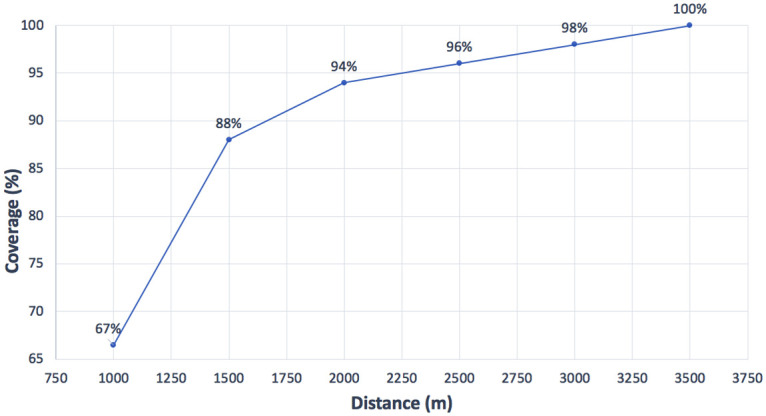
Coverages for the different distances of the 2010 actual schools scenario.

As stated before, 34 primary schools were available in 2010 in Ciudad Benito Juarez. Using the proposed ILP model with *P* = 0, and avoiding resizing the current schools, it is possible to calculate the coverage provided by these schools depending on the total distance allowed for travel from students’ homes to their assigned school. It is worth noting that the real coverage could be worse than the value obtained, since the model makes the optimal assignments student- school and it could happen that bad decisions in the past have led to worse assignments in the reality. Nevertheless, the aim of this first step is just to understand the potential of the initial scenario, without including more resources. Thus, Fig 2 depicts the coverage calculated for each maximum distance *D*_*max*_, i.e., 1000, 1500, 2000, 2500, 3000, and 3500 meters. These values have been selected because they represent a rank order of relatively affordable distances to be covered on foot by primary school students. https://www.overleaf.com/project/613f3352e66ba5c694f60bec According to [32, 35], a distance of 500 meters between home and school can be considered as excellent accessibility, between 500 and 1000 meters as good accessibility, between 1000 and 1500 meters as regular accessibility, between 1500 and 2000 meters as low accessibility, and distances above 2000 meters as poor accessibility. As can be verified, full coverage, i.e., 100%, is only obtained for 3500 meters, which is a very long walking distance (poor accessibility). On the opposite side, the shortest distance tested, i.e., 1000 meters, involves a coverage value of 66%, which means that 34% of the students in public primary schools have to walk more than one kilometer to arrive at their assigned school with available capacity.

Since this is not the most appropriate scenario, some policies are necessary to improve the coverage for short distances.

### Analysis of primary school expansions in 2010

One option to improve the coverage could be resizing the current schools in order to include more students within such short distances. Therefore, some tests have been performed to obtain coverages when *P* = 0 and resizing is allowed. We have considered different budgets *B* ranging from 35,000 to 200,000€. These values have been fixed according to the minimum budget for making an expansion and the maximum budget for building a new school or making two of the most expensive expansions. This allows many different combinations of school resizing while keeping the number of changes under a reasonable value.

**Fig 3:**
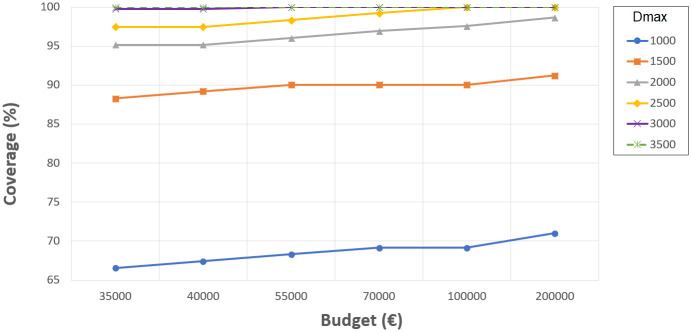
Coverages for the different distances and budgets upon resizing the primary schools in 2010.

Fig 3 provides an overview of the coverages obtained. For 1000 meters, the coverage changed from 66% when resizing was not considered (in previous tests) to the range of 66-71% depending on the investment. For 1500 meters, the previous coverage value obtained without considering resizing was 88%, and with resizing, it ranged from 88 to 91% depending on the investment. For 2000 meters, the previous coverage was 94%, and it changed to 95-98% depending on the investment. For 2500 meters, the previous coverage obtained was 96%, and with resizing, it ranged from 97% to 100%. Finally, for 3000 and 3500 meters, the previous results without resizing were highly satisfactory, and they were always 100% with resizing.

Accordingly, coverages obtained for 1000 and 1500 meters (good and regular accessibility, respectively) do not reach sufficient values when considering resizing and the maximum budget fixed. Nevertheless, larger budgets can be considered, such as the total amount required to expand all schools to the maximum size (about 2,000,000€). This budget is about half the budget invested in building all the schools available in 2010, which is very high. In this case, our experiments indicate that coverages improve, without reaching the 95%. Therefore, new primary schools should be built if coverage of over 95% is desired in terms of these distances.

### Analysis of the location of new schools

Following the conclusions of the previous step, the ILP model has been run using different values of new schools *P*, budgets *B*, and distances *D*_*max*_. The possibility of resizing the current 34 schools is also considered.

**Fig 4:**
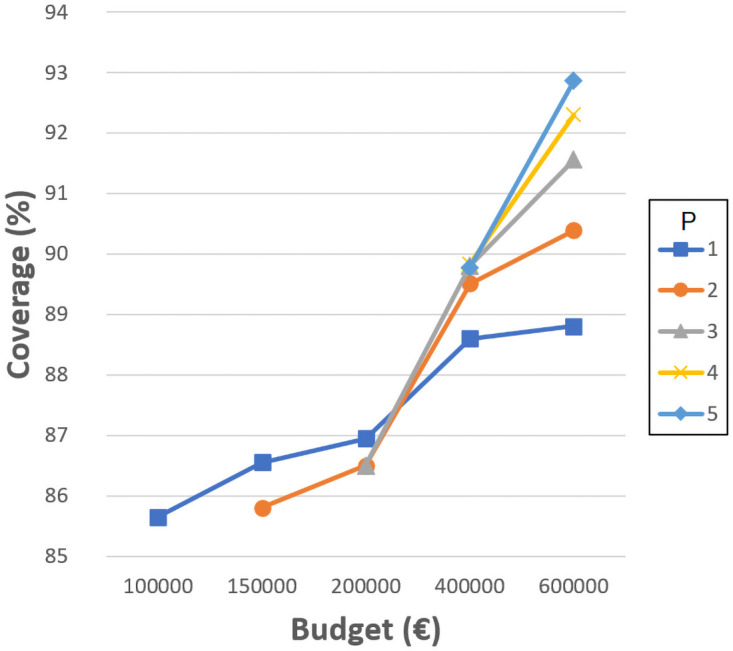
Coverages for 1000 meters.

**Fig 5:**
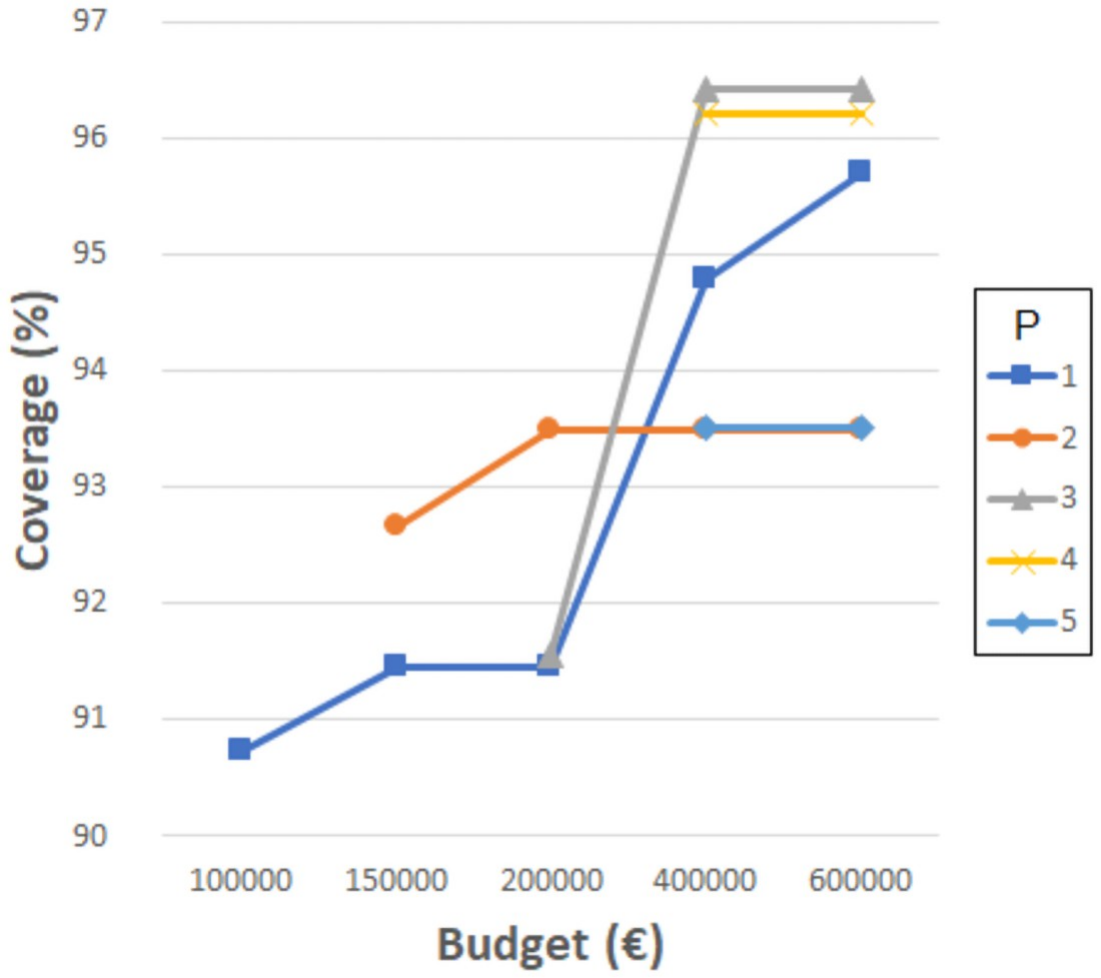
Coverages for 1500 meters.

**Fig 6:**
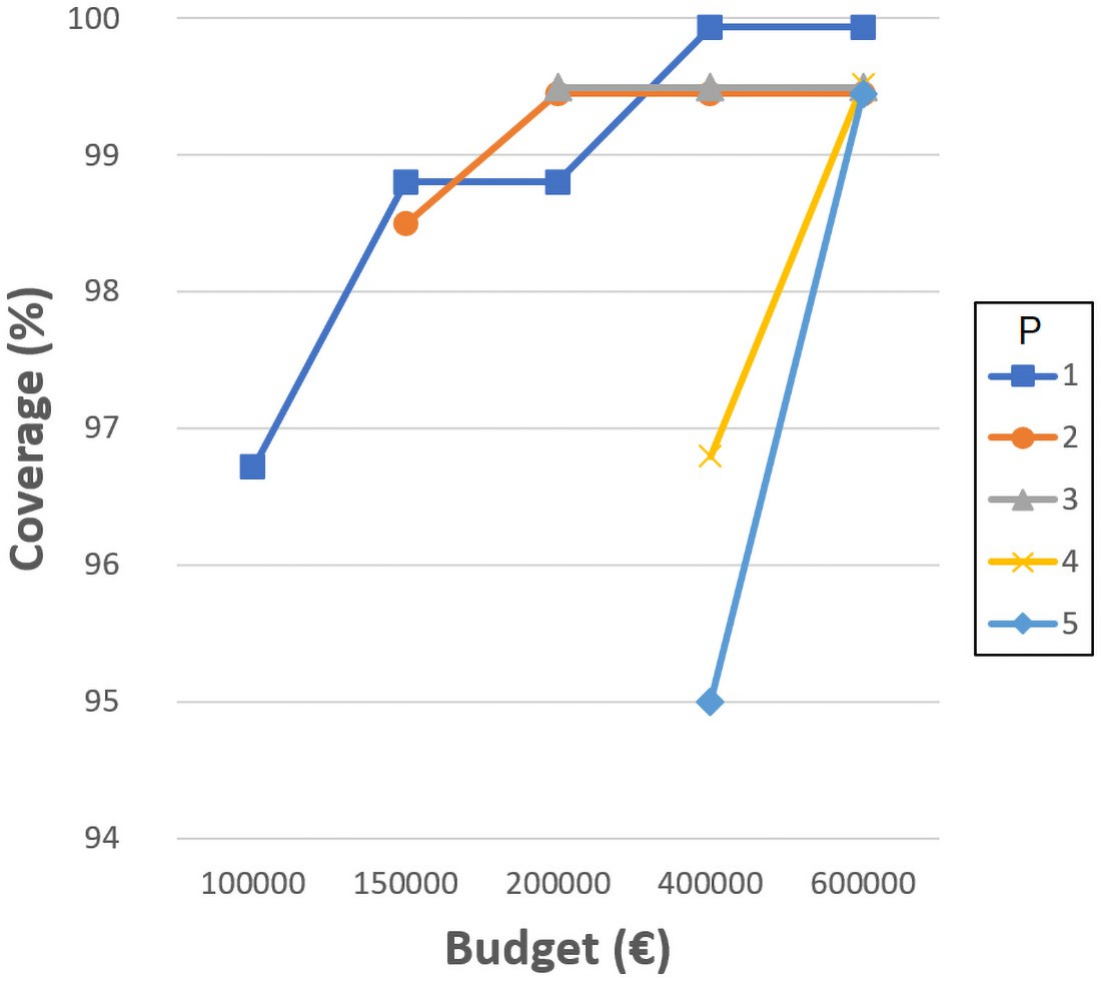
Coverages for 2000 meters.

**Fig 7:**
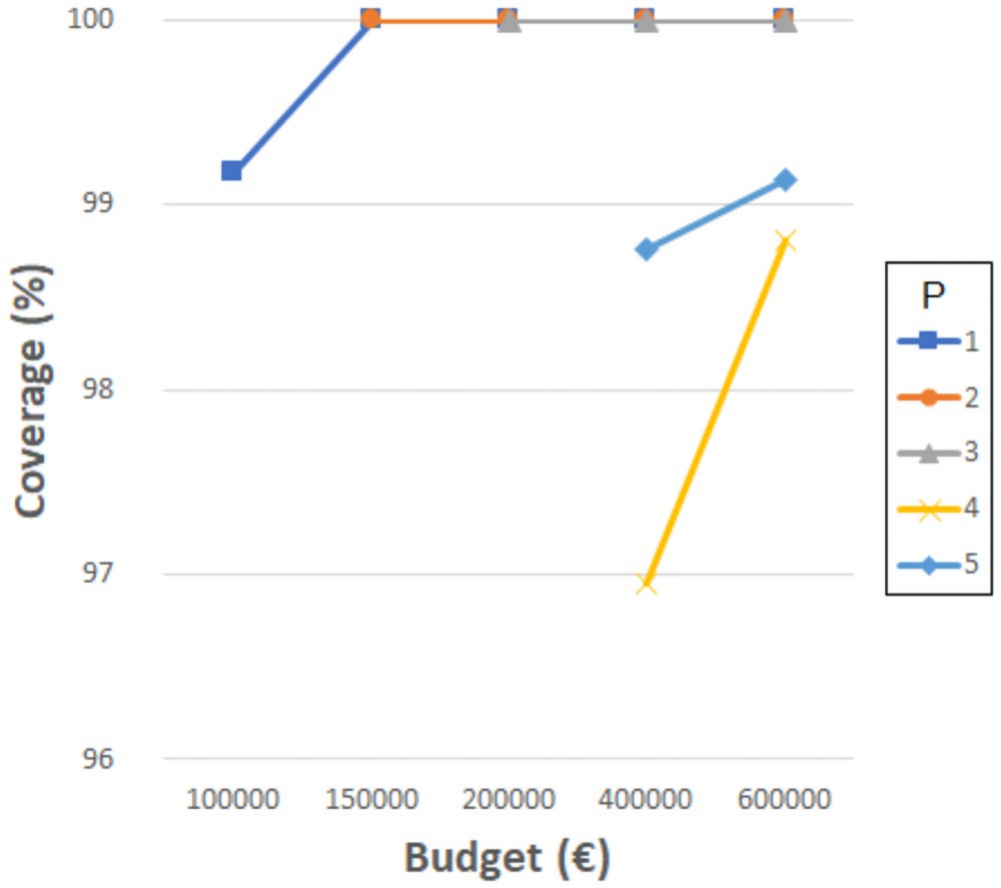
Coverages for 2500 meters.

Figs 4–7 show the the coverage (%) in terms of the budget *B* (€) and the number of new schools to locate *P*. Each of them corresponds to the experiments with *D*_*max*_ 1000, 1500, 2000 and 2500 meters, separately. The remaining distances are not considered in this experiment since, even without allowing the resizing of the schools, the coverages obtained are very close or equal to 100%. Notice that some points do not exist since the associated budget is not enough to build the corresponding *P* schools. Another effect that can be observed is that sometimes building more schools involves worse coverage than building less schools and spending more budget in expansions.

As can be verified, when 1000 meters is considered as the maximum distance, the coverage reaches a maximum value of about 93% when using the highest budget, five new schools, and three expansions. This means that between 14 and 20% (depending on the budget invested) is gained compared to the the previous experiment where only resizing was considered, while new schools were not allowed. However, it is not possible to reach 100% with the attempted number of new schools and budget. Later experiments have elucidated that at least 10 new schools, more than 10 school expansions and a budget of more than 2,000,0000€ are needed to achieve full coverage with *D*_*max*_ 1000 meters. Considering 1500, 2000 and 2500 meters, the maximum coverage values reach 96% (gaining 5%), 99% (gaining 1%) and 100% (gaining 0%), respectively. These last values are high enough to be appropriate, although the distances for students to travel are quite long.

It seems quite clear that the most challenging maximum distance is 1000 meters, although it is also one of the most appropriate. It is worth mentioning that, in some cases, a slight coverage decrease is noticeable when four or five new schools are considered; this is because the budget invested is the same, yet more schools have to be built, so they are smaller and the coverage is affected.

These kinds of graphics could help a decision maker to choose the number of new schools to build according to the budget she/he is willing to invest and the coverage that she/he is required to obtain. Notice that the ILP model also indicates the locations where the new schools must be built, their sizes, and the expansions needed.

### Analysis of optimal primary school locations in 2010 without existing schools

After the previous analysis, some questions arise regarding the locations of the original 34 primary schools. Why are they in those locations? How have responsible staff decided their locations? How does this original distribution affect the coverages? To assess the quality of the distribution of schools in 2010, we can compare it with the optimal solution obtained using the ILP model when an empty scenario is considered. Accordingly, the ILP model has been used while taking into account that the city has no schools, 34 schools need to be located, i.e., *P* = 34, the maximum distance to travel by students should be 1000 meters, i.e., *D*_*max*_ = 1000, and the budget available *B* is the same quantity invested in the original 34 schools (3,640,000€).

When these conditions are applied, the relative gap reported by CPLEX in one hour is slightly high (6%), which means that the model requires more time to obtain the optimal solution. After one day of execution, the optimal solution has been found. The coverage obtained is 99% (almost full coverage), which is well above the original coverage (66%) for this maximum distance *D*_*max*_ = 1000 and higher than the coverages obtained after resizing the current some schools (maximum 71%) and adding new schools with an additional budget (maximum 93%). Consequently, the benefits of using this model are clear. We can conclude that this tool is useful for making decisions not only in given scenarios that contain facilities but also in empty scenarios such as those found when natural disasters occur and it is necessary to develop schools all over again.

**Fig 8:**
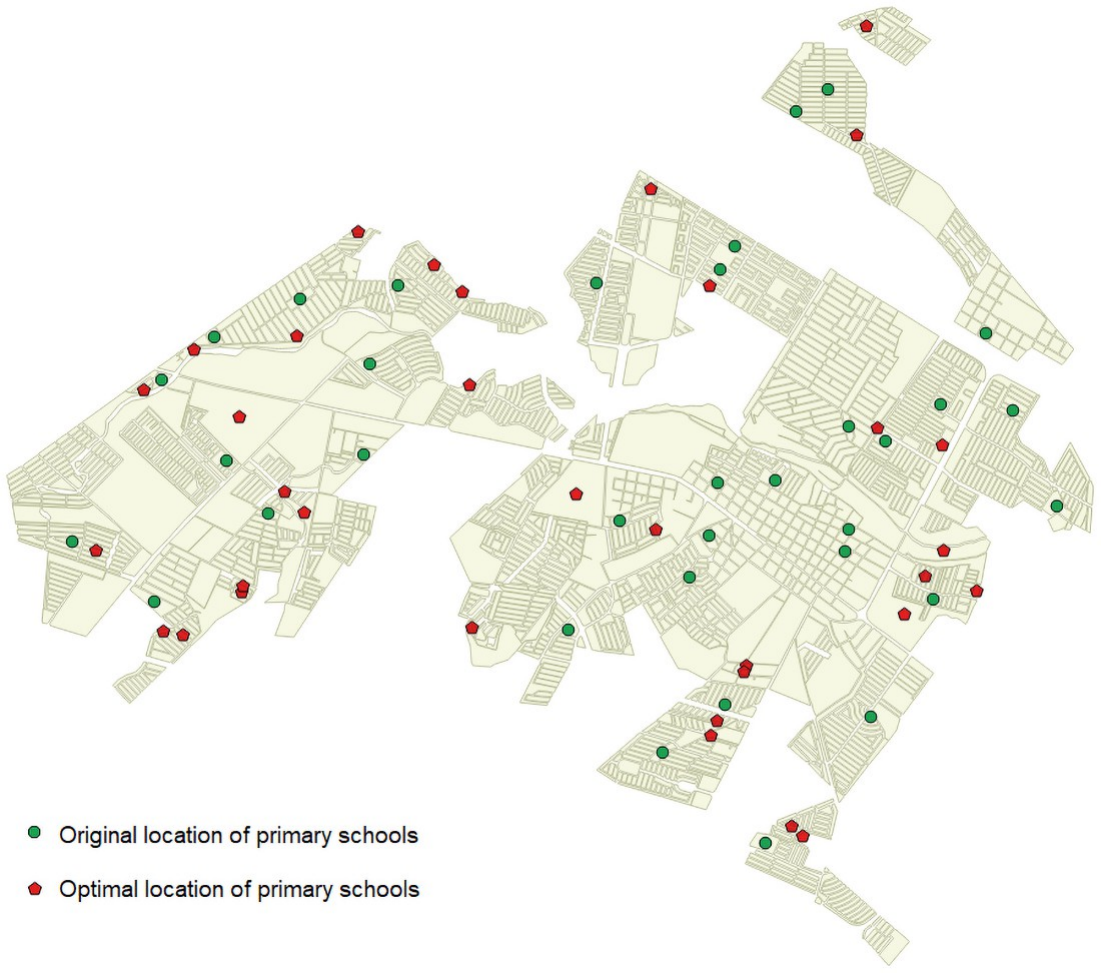
Comparison of original locations of schools and optimal locations of schools.

**Fig 9:**
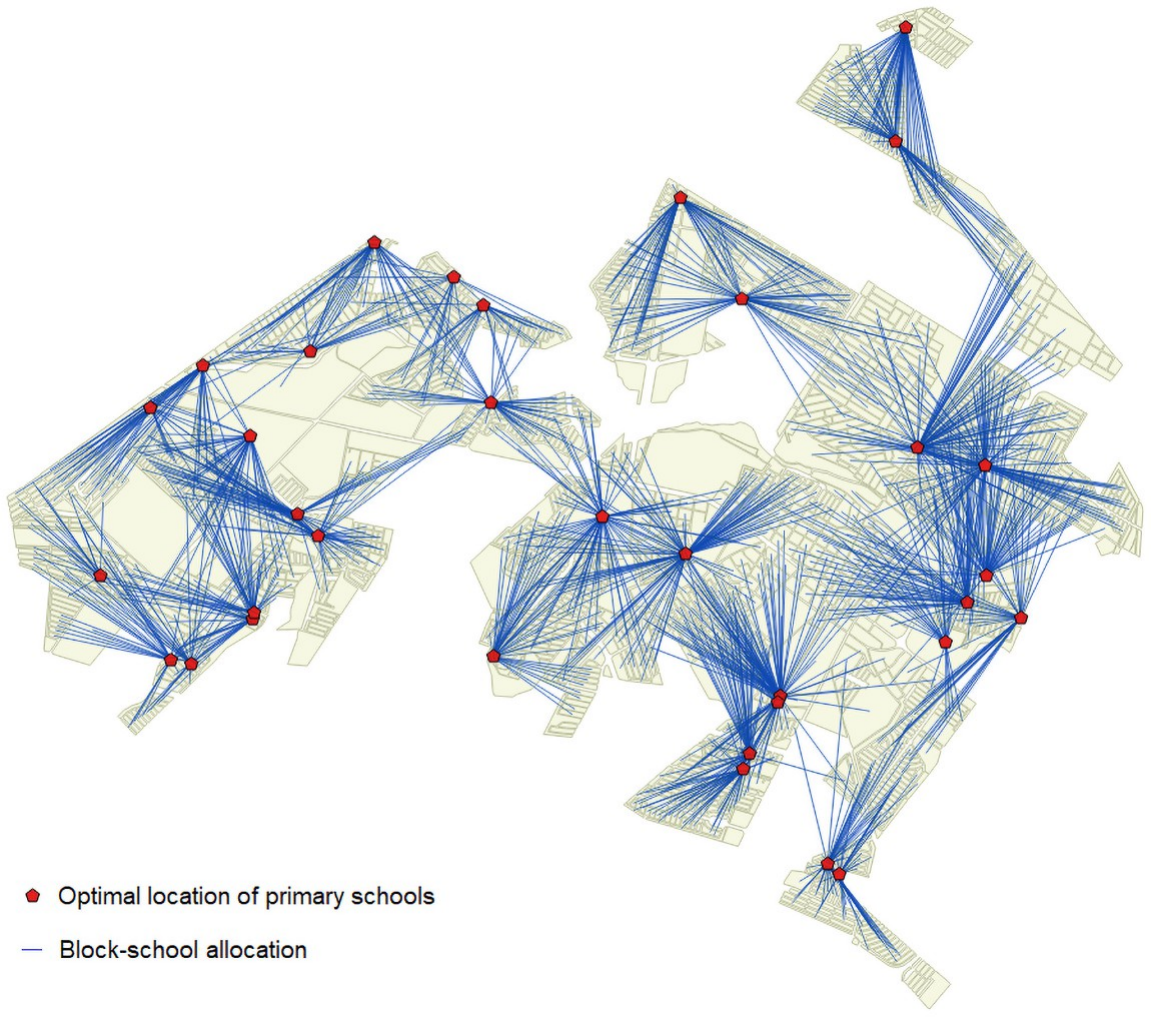
Block-school allocation.

Fig 8 shows the original locations of the 34 primary schools and the optimal locations of these 34 schools through the model with *D*_*max*_ = 1000. Some visual differences are apparent. The original schools are more concentrated in the central-east part of the city, while the locations obtained using the ILP model are more scattered throughout the territory. Fig 9 shows the allocation of blocks to schools. Blocks that are not allocated do not have students or are not covered.

An index of ‘optimality’ can be established by comparing the current coverage of schools in an area (*current*_*coverage*) and the coverage obtained with our model (*optimal*_*coverage*), for example, by using *current*_*coverage*/*optimal*_*coverage*. The values of this index vary from 0 to 1, so the closer to 0 the value is, the worse the current scenario is. Thus, we can obtain a measure of how close a certain scenario is to the best scenario. In this case, the index is 66/99 = 0.66.

As explained before, the proposed methodology will help politicians and public service managers to make better decisions based on data evidence as well as make the decision process more transparent and efficient. Additionally, some knowledge can be extracted by comparing the current locations with the proposed locations. If this information is complemented with historical and political data, an analysis of the reasons that led to the current scenario can be derived. However, such analysis is outside of the scope of this work.

### Projections and primary school location analysis for the future

**Fig 10:**
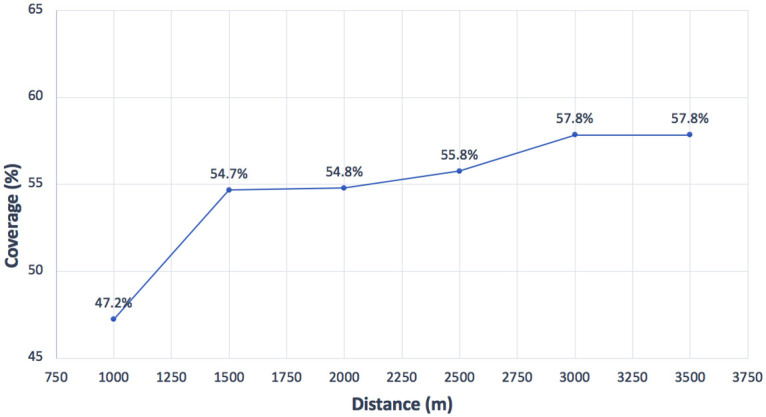
Coverages for the different distances in 2030 considering primary schools in 2010.

**Fig 11:**
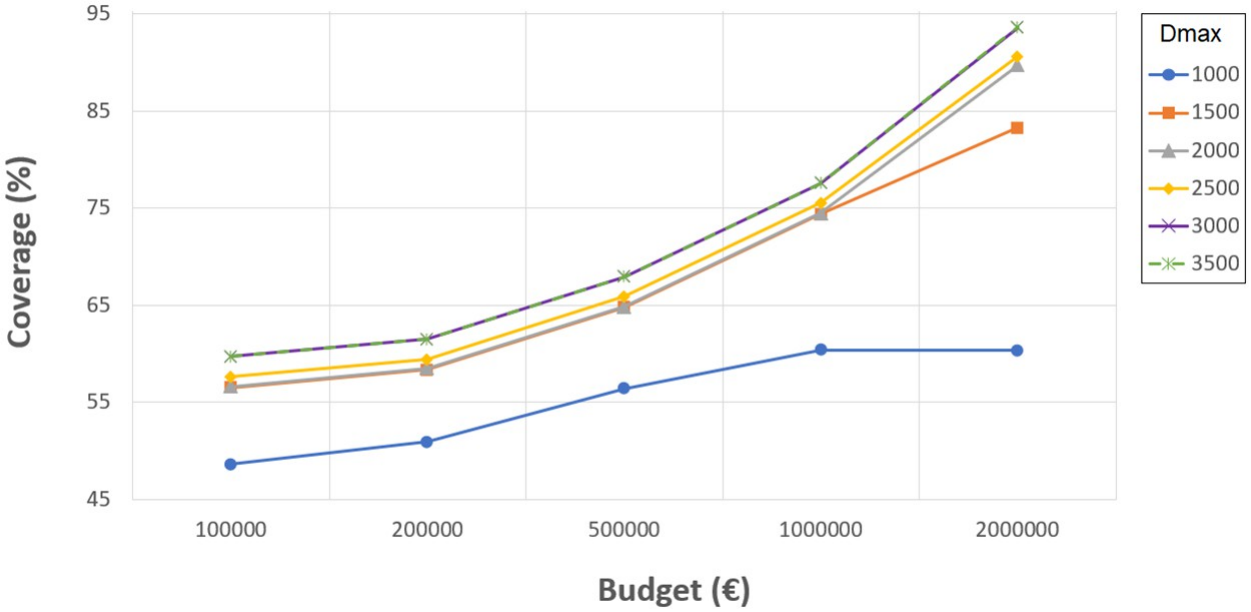
Coverages for the different distances and budgets in 2030 while expanding the primary schools in 2010.

According to the official projections [20], a sharp population increase (80%) is expected from 2010 to 2030. Notice that once the projection of new students has been calculated (as explained in previous section), it is obvious that the schools in 2010 are not sufficient to accommodate the increasing number of students. Even if the expansion of every current school in 2010 is allowed, the resulting total capacity is lower than that needed in 2030. Nevertheless, as previously tested for 2010, the coverages have been calculated when considering the 34 original schools and no resizing allowed. Thus, Fig 10 shows the coverages calculated for each maximum distance *D*_*max*_, i.e., 1000, 1500, 2000, 2500, 3000, and 3500 meters. In this case, the maximum coverage obtained is 57% for 3500 meters, which is quite low, as expected. Next, if resizing is allowed and we consider that every school can be resized (which is unrealistic), then we can calculate the coverages, also with the knowledge that the capacity is inadequate. In this case, the budgets taken into account are higher than those used in the 2010 experiments due to the possibility of resizing every school. Fig 11 depicts the coverages obtained. As can be observed, even with the largest budgets invested, the coverage cannot reach the values of 65% for *D*_*max*_ = 1000 and 85% for *D*_*max*_ = 1500. For the remaining distances, the value of 95% is not reached. Consequently, the need for building new schools is quite clear, especially with the knowledge that allowing the resizing of every school is unrealistic.

To analyze the decisions that should be made in this time period, we have divided it into four periods of five years each (2010-2015, 2015-2020, 2020-2025 and 2025-2030). Thus, we assess the continuous changes required in the primary school system under the assumptions explained in previous section. Notice that in previous analysis with the original data from 2010 (Fig 2), we have obtained a very low coverage (67%) when the maximum walking distance is 1000 meters. As mentioned before, this walking distance is the most appropriate value. Therefore, our aim during each time period is to improve the coverage at this distance. We can set 90% as the desired value, which is an ambitious goal. It is worth mentioning that the decision maker can set this goal to any other value according to expectations.

**Fig 12:**
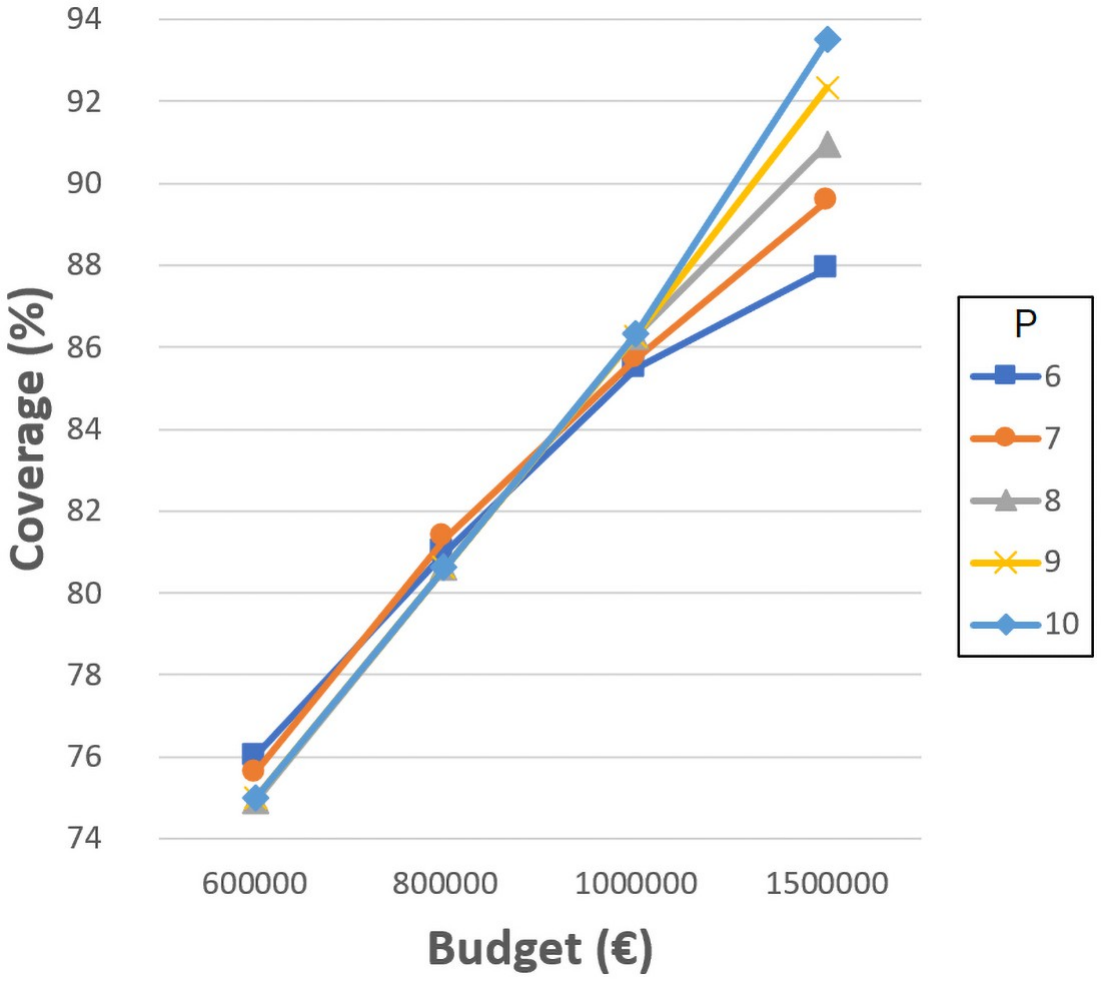
Coverage in 2010-2015 with new schools.

Hence, it is clear that during the first period of 2010-2015, the investment and effort should be high in order to reach this coverage value when starting from 67%. We have tested different budgets and numbers of schools to achieve the goal. Fig 12 shows the results obtained. The lowest budget that allows the target coverage to be achieved is 1,440,000€, with seven new schools. Table 2 shows a summary of the changes needed in this period. The first two rows show the number of schools to be built or expanded and the budget required. The last two rows indicate the distribution of these schools according to size or resizing. As can be verified, the number of schools to build and resize is high, as expected due to the initial scenario.

**Table 2:** Updates in the 2010-2015 period.

	Build				Resize		
Number of schools	7				9		
Budget	930000				510000		
School size	400	600	800	1200	600 → 800	600 → 1200	800 → 1200
Number of schools	0	1	3	3	3	4	2

**Fig 13:**
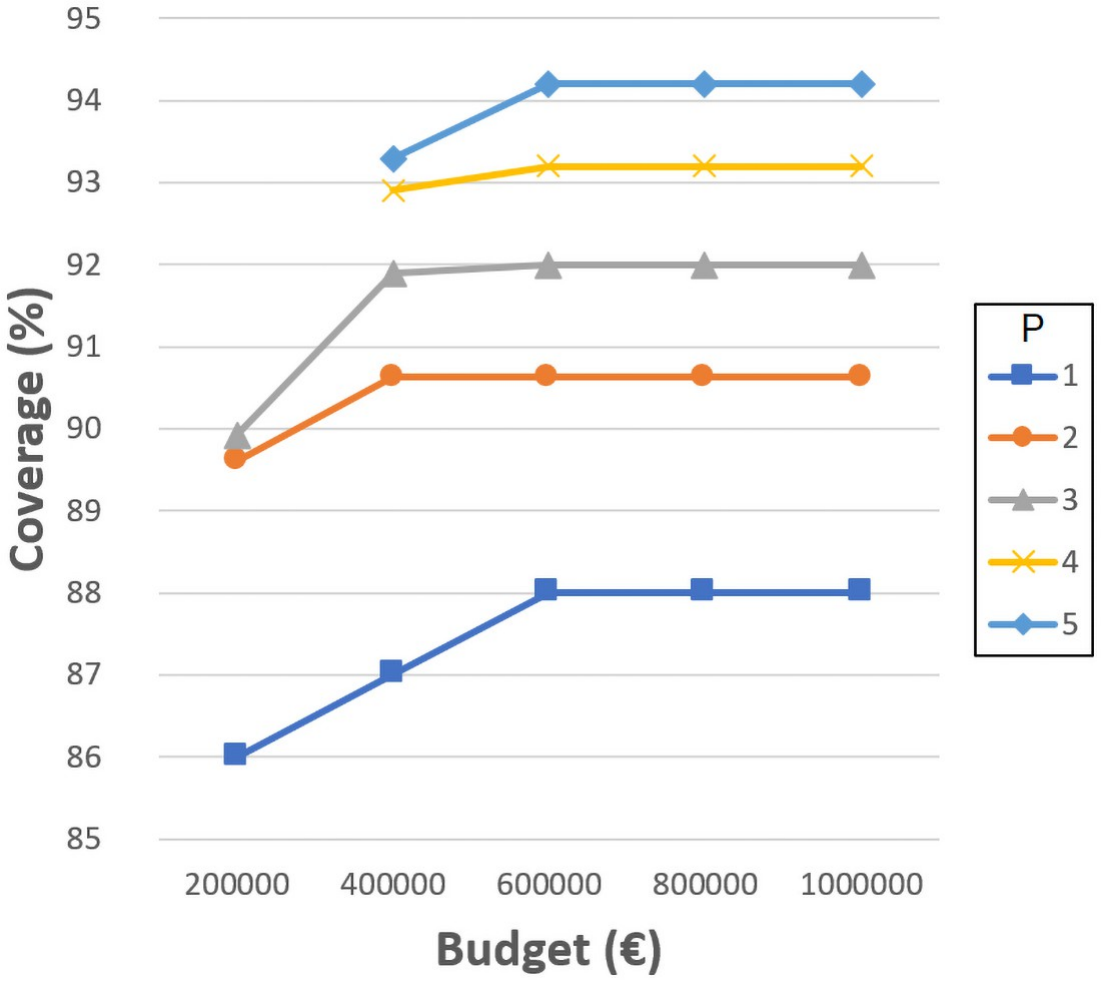
Coverage in 2015-2020 with new schools.

**Fig 14:**
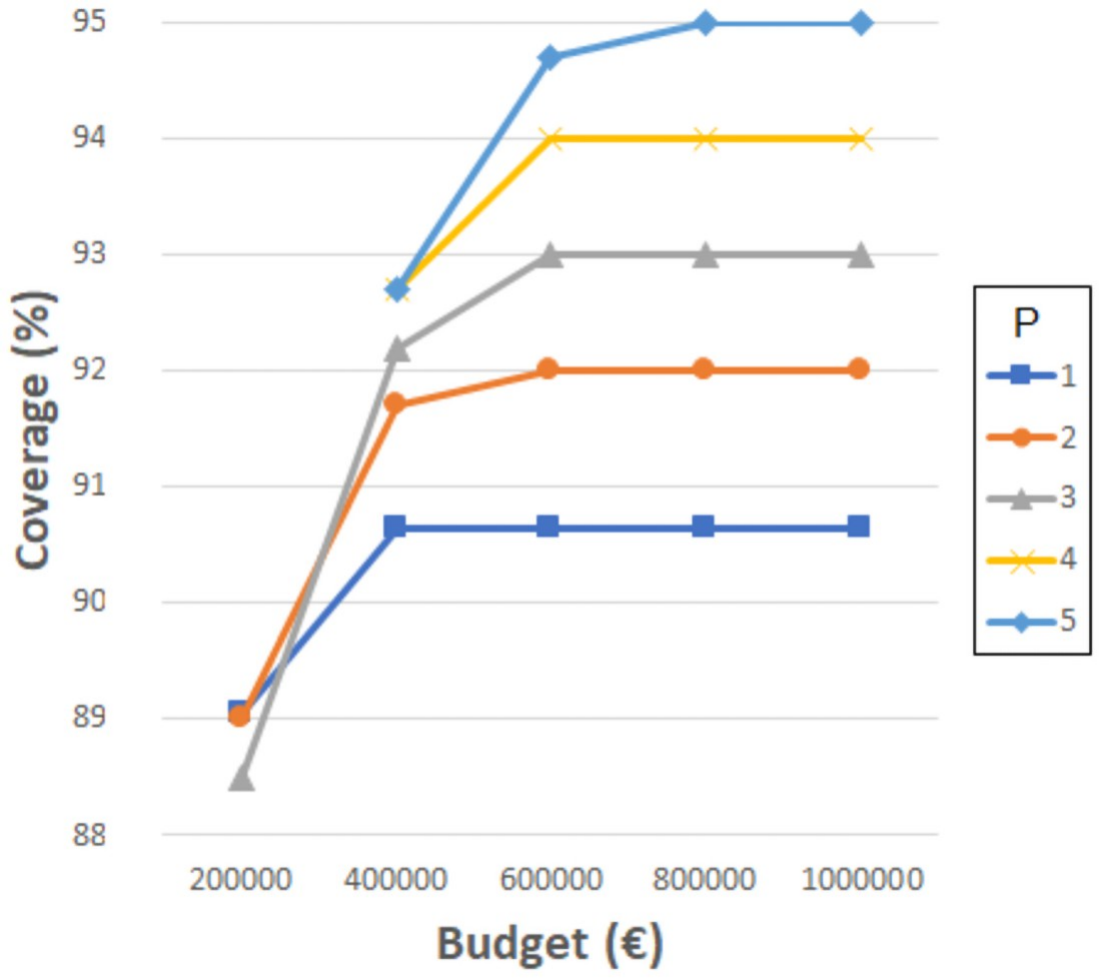
Coverage in 2020-2025 with new schools.

**Fig 15:**
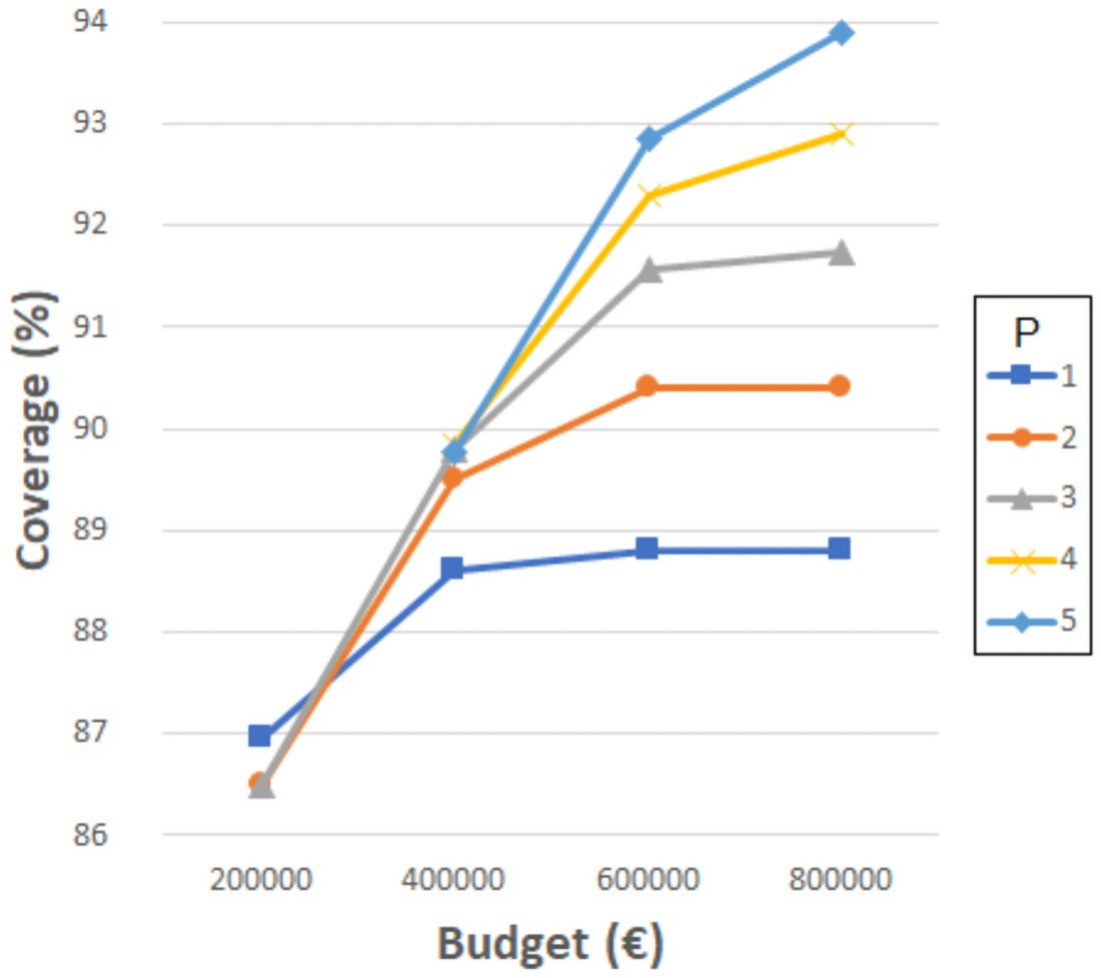
Coverage in 2025-2030 with new schools.

However, if these indications are followed, then the next periods are expected to be less demanding, since the initial scenarios will have better coverage than 2010. Thus, we have developed the same tests for the next period, 2015-2020, and Fig 13 shows the results obtained. In this period, it is possible to achieve the target coverage by building two new schools and investing 375,000€. Table 3 shows a summary of the changes needed in this period. Fig 14 shows the results obtained for the later period of 2020-2025. In this case, the target coverage is reached with one new school, and again, the investment required is 375,000€, although the distribution of this budget is completely different since more expansions are required. Table 4 shows the summary. Finally, Fig 15 shows the results obtained for the last period, 2025-2030. The lowest budget that allows the target coverage to be achieved is 585,000€, with four new schools. Table 5 shows the summary. As can be determined, there is no pattern regarding the changes required throughout time periods. The only common point is that schools of the smallest size are never built, and resizing to the maximum capacity is more common.

**Table 3:** Updates in the 2015-2020 period.

	Build				Resize
Number of schools	2				3
Budget	210000				165000
School size	400	600	800	1200	800 → 1200
Number of schools	0	1	1	0	3

**Table 4:** Updates in the 2020-2025 period.

	Build				Resize			
Number of schools	1				5			
Budget	120000				255000			
School size	400	600	800	1200	400 → 600	600 → 800	600 → 1200	800 → 1200
Number of schools	0	0	1	0	1	1	1	2

**Table 5:** Updates in the 2025-2030 period.

	Build				Resize
Number of schools	4				3
Budget	420000				165000
School size	400	600	800	1200	800 → 1200
Number of schools	0	2	2	0	3

**Fig 16:**
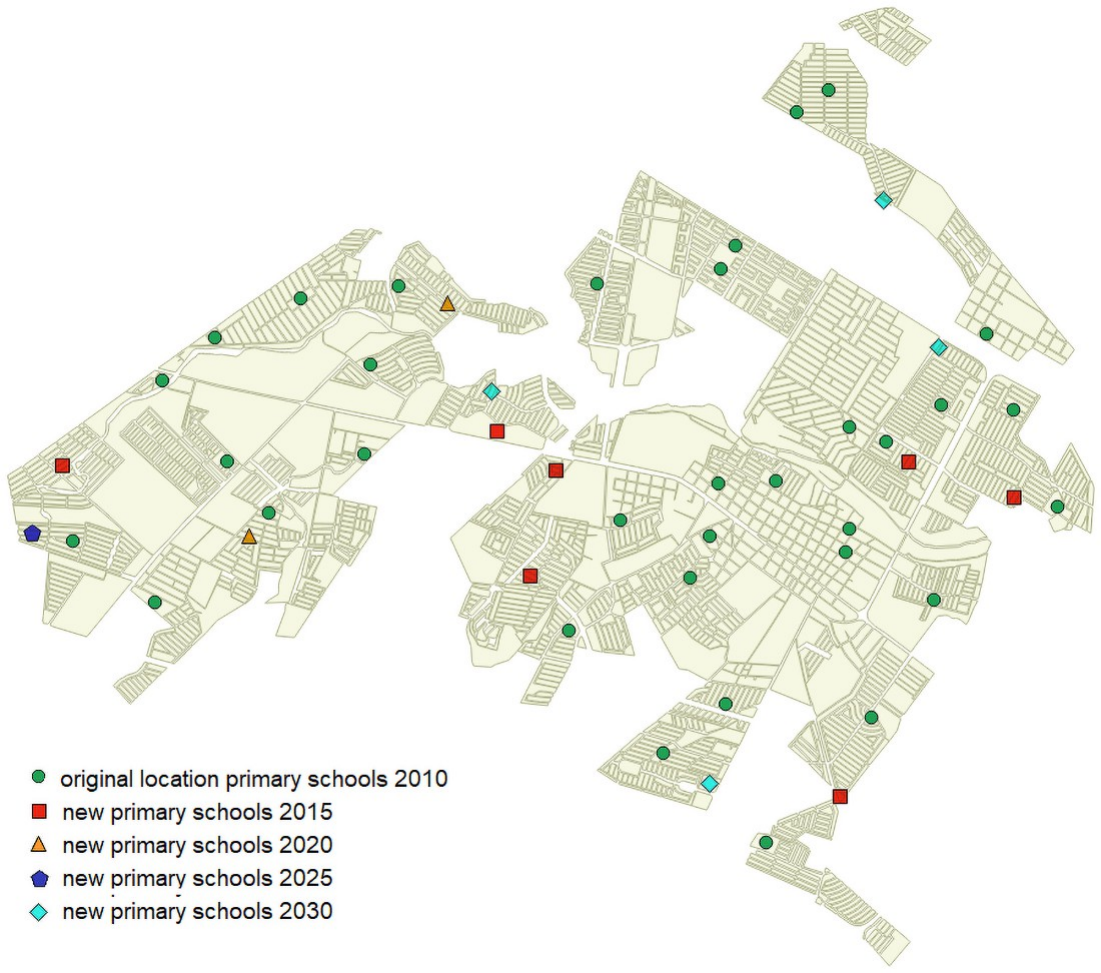
Location of the new schools throughout time periods.

Fig 16 shows the locations of the initial schools and the locations of the new schools required to fulfill the target coverage. During the first period, the new schools are scattered, reinforcing the initial network that presented bad coverage at the distance desired. In the next two periods, the focus is on the western part of the city. Finally, the peripheral areas of the rest of the city are reinforced with new schools.

This could be an initial plan for the whole period that provides knowledge of the future investments required, the future location of new primary schools and expansions of the current schools.

Notice that a source of uncertainty in general location models is the demand. However, it is not common to address this point in the literature about location of schools. In this regard, we have developed a sensitivity analysis to find out how the solutions obtained are adapted to different scenarios.

For each solution obtained corresponding to each time period, we have checked how the coverage is affected if the demand (i.e., number of students) in each census block changes. We have assumed that the deterministic values that we used in previous experiments are the average of a normal distribution. Then, different variances have been considered: low (0.2), medium (0.5) and high (1). Note that we use this probability distribution because it is one of the most common and usually functions well. When enough details about the evolution of the population in each block were available, we were able to obtain the probability distribution that best fit with these data.

Thus, we have generated one scenario for each variance and each time period. Table 6 shows the results of this analysis. The first column corresponds to the coverage obtained without uncertain demand, i.e., previous results. The next three columns shows the coverage obtained under low, medium and high variances. As can be verified, the coverage is practically not affected by these changes in the demand. Although more scenarios for each variance should be tested to conclude that the deterministic results are robust with respect to demand uncertainty, this initial study seems to indicate that they are. Another option is to consider the uncertainty in the solution approach, with the aim of obtaining the most robust solutions. In this regard, we propose to integrate simulation with a metaheuristic (i.e., simheuristics [53]) as further work.

**Table 6:** Coverage under different scenarios of demand.

	Deterministic	Low variance	Medium variance	High variance
2010-2015	90.56	90.39	90.40	90.30
2015-2020	90.63	90.48	90.47	90.48
2020-2025	90.63	90.59	90.58	90.61
2025-2030	92.30	92.20	92.24	92.13

## Conclusions

Education is fundamental in development and growth. One relevant issue with respect to access to education is the availability of schools within a reasonable distance. This research contributes to improving the accessibility to schools by presenting an ILP mathematical model and a framework of analysis that can be used to help decision makers in these endeavors. The model determines the location and size of new schools, as well as the resizing of current schools, so that the coverage at a certain established home-school distance is optimized under budget constraints. The framework provides a set of steps to develop the analysis of the location of schools. We focus on relatively poor areas with a sharp population increase tendency and where public schools are equally attractive: with the same quality, similar teaching, recreation spaces, tradition, and pupils from the same social stratum. In such areas, walking is the most common means of traveling from home to school, and therefore, the objective is equity oriented in terms of accessibility. Thus, the modeling employed assumes that students walk to school and therefore tend to prefer the school closest to home.

The model and the framework are applied to the location of public primary schools in Ciudad Benito Juarez as a case study, although similar challenges are faced by most developing countries. In general, the methodology is highly flexible, making it possible to use it to analyze different scenarios, according to the policies to be implemented.

According to the framework, we have first checked the coverage of schools in 2010, and we have verified poor accessibility: 33% of the students must walk more than 1000 meters from home to their assigned school with enough capacity, and 12% must walk more than 1500 meters. In the second step, when new schools are not considered, but expansions are one possible option, it has been verified that the coverage obtained with schools in 2010 can be improved, although it is still below the desired values. In the third step, when new schools and resizing are considered, the coverage can reach quality values for almost every tested distance. However, at least 10 new schools are required to obtain good coverage for the 1000 meters, which is considered to be good accessibility. This result leads to the conclusion that the original distribution of schools over the area is not appropriate and has not considered the right parameters. To prove this conclusion, we have continued to the fourth step. We have used the same budget and number of schools present in 2010 and have run the model while taking into account that the area does not contain any school. The results have demonstrated that a considerably better school network can be developed when our proposed model is employed, moving from a coverage value of 67% to 93% for the distance of 1000 meters.

Following the last step of the framework, we have analyzed scenarios in the future using some projections. We have considered four periods of five years each from 2010 to 2030. A target coverage must be set for each period, and in this case study, we fix it as 90% for a *D*_*max*_ of 1000 meters. We have determined that the first period is the most difficult since the initial locations of schools provide only low coverage for this walking distance. These circumstances and the projected increase in population involve the requirement for seven new schools scattered over the city, resizing of nine schools and a large budget. In later periods, the budget and number of schools to add and resize are lower. In this case, the location of new schools basically reinforces some parts of Ciudad Benito Juárez, such as the west and peripheral areas. Finally, we have checked the robustness of the solutions for each time period when demand uncertainty is considered. We have created different scenarios with different levels of demand variation, and we have determined that the coverage remain almost unchanged.

We can conclude that the proposed model and framework can help politicians and public service decision makers to evaluate different scenarios in relatively poor areas with sharp population increase tendencies in a transparent and efficient way. Consequently, the work will positively impact society. Different specializations and even the community should participate in the development of the input data to avoid illogical results, for example, proposing to build a school in a protected area or very close to inappropriate places such as a prison or sex offenders. However, the flexibility offered by our approach makes it possible to change input data and some constraints to easily consider different scenarios.

As future research, it is expected that the proposed model and framework will be expanded to consider the singularities of different levels of schools (primary, high school), school buses or other means of transportation, security and safety issues, environmental issues, etc. Additionally, it would be relevant to develop an approach that intrinsically considers the uncertainty. In this regard, we will design and implement a simheuristic approach combining simulation and a heuristic or metaheuristic. This solution approach can be included in a decision-making tool to help the decision maker to evaluate different scenarios.
